# Cardiovascular risk profile and frailty in a population-based study of older British men

**DOI:** 10.1136/heartjnl-2014-306472

**Published:** 2014-12-05

**Authors:** S E Ramsay, D S Arianayagam, P H Whincup, L T Lennon, J Cryer, A O Papacosta, S Iliffe, S G Wannamethee

**Affiliations:** 1 Department of Primary Care & Population Health, UCL, London, UK; 2 Population Health Research Institute, St George's University of London, London, UK

## Abstract

**Background:**

Frailty in older age is known to be associated with cardiovascular disease (CVD) risk. However, the extent to which frailty is associated with the CVD risk profile has been little studied. Our aim was to examine the associations of a range of cardiovascular risk factors with frailty and to assess whether these are independent of established CVD.

**Methods:**

Cross-sectional study of a socially representative sample of 1622 surviving men aged 71–92 examined in 2010–2012 across 24 British towns, from a prospective study initiated in 1978–1980. Frailty was defined using the Fried phenotype, including weight loss, grip strength, exhaustion, slowness and low physical activity.

**Results:**

Among 1622 men, 303 (19%) were frail and 876 (54%) were pre-frail. Compared with non-frail, those with frailty had a higher odds of obesity (OR 2.03, 95% CI 1.38 to 2.99), high waist circumference (OR 2.30, 95% CI 1.67 to 3.17), low high-density lipoprotein-cholesterol (HDL-C) (OR 2.28, 95% CI 1.47 to 3.54) and hypertension (OR 1.79, 95% CI 1.27 to 2.54). Prevalence of these factors was also higher in those with frailty (prevalence in frail vs non-frail groups was 46% vs 31% for high waist circumference, 20% vs 11% for low HDL and 78% vs 65% for hypertension). Frail individuals had a worse cardiovascular risk profile with an increased risk of high heart rate, poor lung function (forced expiratory volume in 1 s (FEV_1_)), raised white cell count (WCC), poor renal function (low estimated glomerular filtration rate), low alanine transaminase and low serum sodium. Some risk factors (HDL-C, hypertension, WCC, FEV_1_, renal function and albumin) were also associated with being pre-frail. These associations remained when men with prevalent CVD were excluded.

**Conclusions:**

Frailty was associated with increased risk of a range of cardiovascular factors (including obesity, HDL-C, hypertension, heart rate, lung function, renal function) in older people; these associations were independent of established CVD.

## Introduction

The increasing numbers of older people in countries such as the UK poses a significant public health challenge to improving health and social care outcomes in older populations.^1^ A particular concern in older people is the development of frailty, defined as “a clinically recognizable state of increased vulnerability, resulting from aging-associated decline in reserve and function across multiple physiologic systems such that the ability to cope with everyday or acute stressors is compromised”.^2^ The reduced resilience of frail individuals to stressors leads in turn to an increased vulnerability to adverse outcomes, including functional decline, hospitalisation, disability, long-term care and death.^3–5^ Frailty therefore represents one of the greatest challenges for healthcare professionals in countries with ageing populations such as the UK.^3^ A systematic review reported the prevalence of frailty as between 4% and 59% (varying due to the varying methods of measuring frailty) in studies with participants aged ≥65 years.^6^

Apart from disability and mortality, frailty is also associated with an increased risk of cardiovascular disease (CVD). A systematic review showed that frailty was associated with a twofold to threefold increase in risk of CVD.^7^ Frailty is also a strong predictor of mortality in patients with CVD independent of age, disease severity, comorbidity and disability.^7^ Previous studies have attempted to understand the pathophysiology underlying this collinear relationship between frailty and CVD—factors that have been found to play a role underlying this relationship include inflammation, and other risk factors for CVD and mortality such as chronic kidney disease and low alanine transaminase (ALT).^8–11^ However, few population-based studies have described the cardiovascular risk profile associated with frail older people.^8^ ^9^ This is particularly important since a better understanding of the cardiovascular risk profile of frail older adults will enable better clinical management to reduce the high risk of CVD associated with frailty. Indeed, the identification and management of frailty is increasingly being recognised as a potentially important aspect of the overall care of older patients with CVD.^7^ Furthermore, little is known about whether an adverse cardiovascular risk profile is present in frail older adults, even in the absence of established CVD. Therefore, the aim of this study was to examine whether frailty was associated with a range of cardiovascular risk factors in a community-dwelling study of older British men and to investigate whether the associations are present in those without established CVD.

## Methods

The British Regional Heart Study is a prospective study of CVD comprising a socially and geographically representative sample of 7735 men aged 40–59 years from one general practice in each of 24 towns representing all major British regions and who were initially examined in 1978–1980.^12^ In 2010–2012, all surviving men (n=3137) now aged 71–92 years were invited to attend a 30-year re-examination. The men were requested to fast for a minimum of 6 h and to attend a measurement session at a specified time between 08:00 and 18:00. Participants underwent a physical examination, provided a fasting blood sample and completed a questionnaire (at the time of examination or by post if they did not attend) providing information on their medical history and lifestyle factors. Presence of comorbidity was based on subjects’ reporting of a doctor diagnosis of the following conditions reported in the questionnaire—history of angina, heart attack, heart failure, high blood pressure, stroke, peripheral vascular disease (PVD), deep vein thrombosis (DVT), diabetes, chronic kidney disease, anaemia, asthma, bronchitis, arthritis, falls, cataract and depression.

Physical examination of subjects involved anthropometric (height, weight, waist circumference) and physiological (blood pressure, lung function) measurements. A 12-lead ECG was recorded. Height and weight were measured in subjects in light clothing and without shoes. Height was measured with a Harpenden stadiometer to the last complete 0.1 cm and weight with a Tanita MA-418-BC body composition analyser (Tanita, Tokyo, Japan). Waist circumference (cm) was measured in duplicate with an insertion tape (CMS, London). Waist measurement was measured from the midpoint between the iliac crest and the lower ribs measured at the sides. Body mass index (BMI) was calculated as weight/(height)^2^ (kg/m^2^). Blood pressure was measured using an Omron blood pressure recorder twice in succession in the right arm, with the subject seated and the arm supported. The mean of the two blood pressure recordings was used in the analysis. Physical performance assessments included a walking test (time taken, in seconds, to walk 3 m at normal walking pace), and grip strength measured with a Jamar Hydraulic Hand Dynamometer. Grip strength (in kilograms) was measured thrice for each hand, and the best of six readings was used for the analysis.

*Blood measurements*: Fasting serum samples were measured for lipids and markers of hepatic and renal function. Total cholesterol, high-density lipoprotein cholesterol (HDL-C) and triglycerides were measured with enzymatic colorimetric assays using methods of Nauck *et al* and Wahlefeld *et al*, respectively.^13^ ^14^ Creatinine,^15^ alkaline phosphatase^16^ (ALP) and hepatic enzymes including ALT^17^ and γ-glutamyltransferase (GGT)^18^ were also measured using enzymatic colorimetric assays. Albumin,^19^ calcium,^20^ magnesium^21^ and phosphate^22^ were measured using endpoint colorimetric assays. Sodium was measured using ion-selective electrode technique.^23^ Glucose was measured in a fluoride oxidase plasma sample.^24^ Impaired fasting glucose was taken as >6.1 and <7 mmol/L. Estimated glomerular filtration rate (eGFR), as a measure of renal function, was estimated from serum creatinine using the modification of diet in renal disease equation developed by Levy *et al*.^25^

*Lung function*: Forced expiratory volume in 1 s (FEV_1_) was measured as part of lung function tests. Tests were carried out standing and without nose clips. A Vitalograph Compact II instrument was used, which was calibrated at least twice daily using a precision syringe. FEV_1_ was recorded for the best test, defined in accordance with the American Thoracic Society recommendations. Cole has shown that dividing by the height squared is the most appropriate way of standardising lung function for stature.^26^ FEV_1_ was height standardised to the average height, 1.71 m, in the study. Thus, height standardised FEV_1_=FEV_1_ × (1.71/height)^2^. Low FEV_1_ was defined as being in the lowest quartile of FEV_1_.

*Lifestyle factors*: Subjects were asked detailed questions about their smoking and drinking habits. The men were classified into groups based on their alcohol intake—none, occasional, light, moderate and heavy. Heavy drinking was defined as drinking >6 units (1 UK unit=10 g) of alcohol daily or on most days. In the questionnaire, subjects were also asked to report their pattern of physical activity such as walking, cycling and other sporting activities. Physical activity scores were assigned on the basis of frequency and type of activity, and the men were divided into six groups: none, occasional, light, moderate, moderately vigorous and vigorous. Subjects who reported none or occasional activity were classified as ‘inactive’.

*Definitions*: The International Society for Hypertension Guidelines (2003) were used to identify patients with hypertension as with systolic blood pressure ≥160 mm Hg or diastolic blood pressure ≥90 mm Hg or on antihypertensive treatment. Low HDL-C was defined as levels <40 mg/dL (<1.04 mmol/L), and high triglycerides as ≥200 mg/dL (≥2.3 mmol/L).^27^ Low sodium was taken as <138 mmol/L. High creatinine, ALP, GGT and phosphate were taken as the top fifth of the distributions. Low albumin, ALT, calcium and magnesium were taken as the bottom fifth of the distribution. High white cell count (WCC) was taken as the top fifth of the distribution. Anaemia was defined as haemoglobin levels <13 g/dL.

Assessment of frailty was based on the ‘Fried frailty phenotype’^4^ using both questionnaire and objective data. This included unintentional weight loss (assessed as ≥5% decrease in self-reported weight that was reported to be unintentional); exhaustion (if response to the question ‘Do you feel full of energy?’ was ‘no’); weakness (assessed as lowest fifth of grip strength distribution); and slow walking speed (lowest fifth of walking speed). If walking speed was unavailable, self-report of slow walking pace (being unable to walk more than a few steps or <200 yards or difficulty walking across a room) or low physical activity (self-report of being less/much less active than an average man). Presence of three or more of these components was defined as frailty, and presence of one or two as pre-frailty.

### Statistical analyses

Multivariable logistic regression was used to estimate age-adjusted odd ratios (ORs) and 95% confidence intervals (CIs) according to categories of frailty with ‘not frail’ as the reference group. Box and whisker plots were obtained to present the distribution of BMI, waist circumference, HDL-C and systolic blood pressure according to frailty groups. ORs and 95% CI were calculated for lifestyle factors (smoking, alcohol, obesity, waist circumference), chronic diseases and vascular risk factors.

## Results

A total of 1722 men (55%) attended the examination. Questionnaires were completed by 2137 men (68% response rate). Of the 1722 men who attended the examination, 100 men did not have information on measurements for frailty; therefore, the analysis was restricted to 1622 men.

Among 1622 men aged 71–92 years, 303 men (19%) were frail and 876 men (54%) were pre-frail. Of the pre-frail group, 57% had one frailty criteria and 43% had two. In the frail group, 66% had three of the frailty criteria, 30% had four and 4% had five of the criteria. Table 1 describes the demographic and lifestyle factors according to frailty. Men in the frail group were slightly older and less likely to be never smokers compared with those not frail. Moderate/heavy alcohol consumption was not associated with frailty.

**Table 1:** Demographic and lifestyle factors according to frailty in a population-based study of 1622 older British men aged 71–92 years in 2010–2012

	Not frail (n=443)	Pre-frail (n=876)	Frail (n=303)
Age (years)			
Mean (SE)	77 (0.21)	79 (0.15)	81 (0.26)
Never smoker			
n (%)	189 (43%)	331 (38%)	93 (31%)
OR (95% CI)	1.00	0.85 (0.67 to 1.08)	0.65 (0.47 to 0.89)
Moderate/heavy alcohol consumption			
n (%)	20 (5%)	41 (5%)	12 (4%)
OR (95% CI)	1.00	1.16 (0.67 to 2.02)	1.10 (0.52 to 2.33)
Manual social class			
n (%)	196 (45%)	385 (45%)	152 (52%)
OR (95% CI)	1.00	1.04 (0.82 to 1.31)	1.44 (1.05 to 1.96)

All ORs are age-adjusted.

Table 2 presents the associations between frailty and CVD. Men with frailty had greater risks of angina, coronary disease, stroke, PVD, heart failure, DVT and diabetes. Increased risks of these conditions were also observed in the pre-frail group.

**Table 2:** Cardiovascular disease and related chronic conditions according to frailty in a population-based study of 1622 older British men aged 71–92 years

	Not frail	Pre-frail	Frail
Angina			
n (%)	45 (10%)	149 (17%)	80 (27%)
OR (95% CI)	1.00	1.75 (1.22 to 2.50)	2.94 (1.64 to 4.45)
Heart attack			
n (%)	41 (9%)	123 (14%)	63 (21%)
OR (95% CI)	1.00	1.61 (1.11 to 2.35)	2.64 (1.70 to 4.11)
Stroke			
n (%)	23 (5%)	86 (10%)	51 (17%)
OR (95% CI)	1.00	2.00 (1.24 to 3.23)	3.78 (2.22 to 6.45)
Peripheral vascular disease			
n (%)	4 (1%)	36 (4%)	22 (7%)
OR (95% CI)	1.00	5.01 (1.77 to 14.20)	10.01 (3.36 to 29.85)
Heart failure			
n (%)	1 (0%)	16 (2%)	18 (6%)
OR (95% CI)	1.00	8.56 (1.13 to 64.97)	30.93 (4.05 to 236.43)
Deep vein thrombosis			
n (%)	8 (2%)	24 (3%)	18 (6%)
OR (95% CI)	1.00	1.55 (0.69 to 3.50)	3.56 (1.48 to 8.53)
Diabetes			
n (%)	60 (14%)	135 (15%)	83 (27%)
OR (95% CI)	1.00	1.24 (0.89 to 1.73)	2.78 (1.89 to 4.09)

All ORs are age-adjusted.

**Figure 1:**
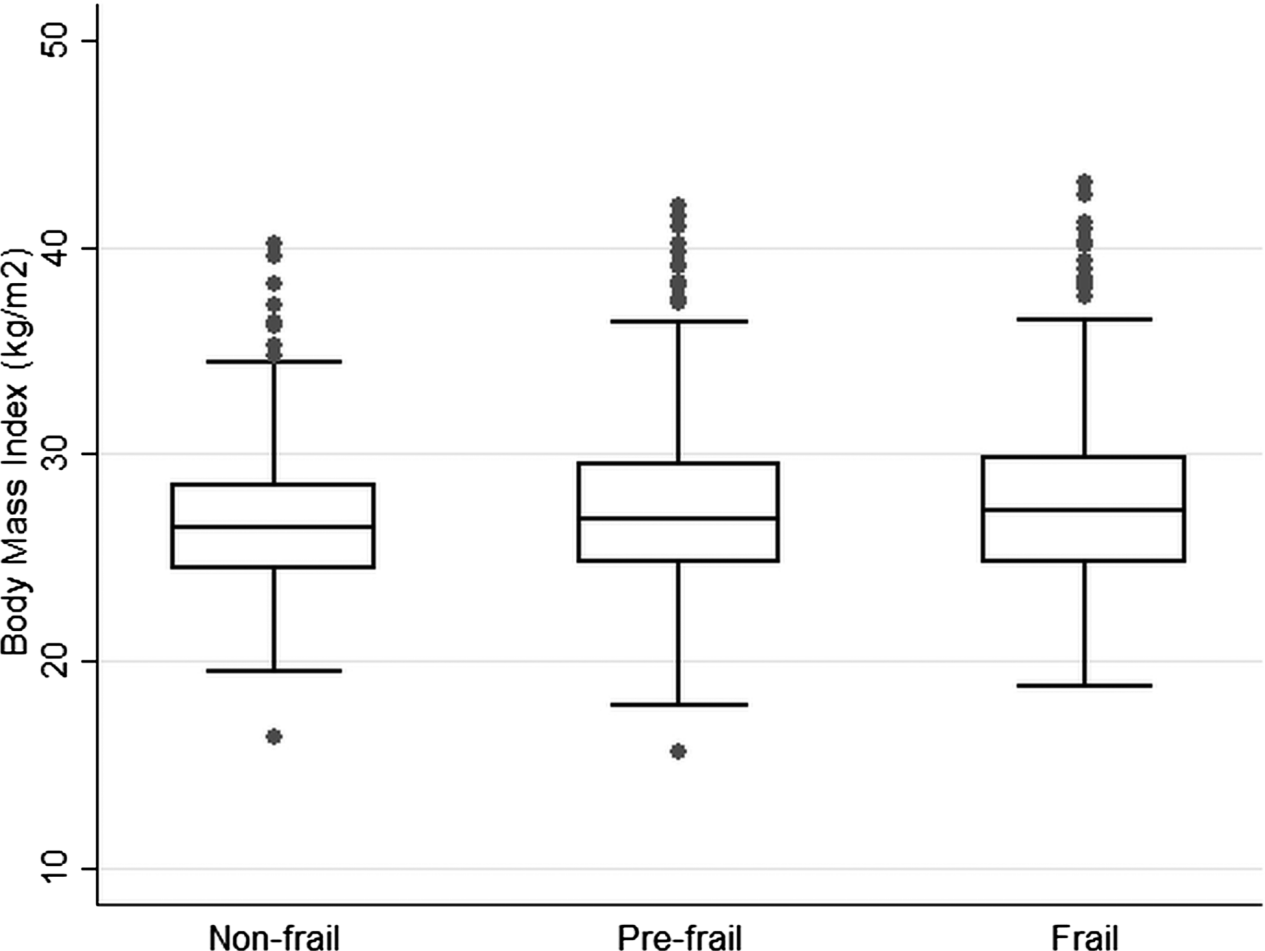
Box and whisker plot with the distribution of body mass index according to frailty groups in 1622 older British men.

**Figure 2:**
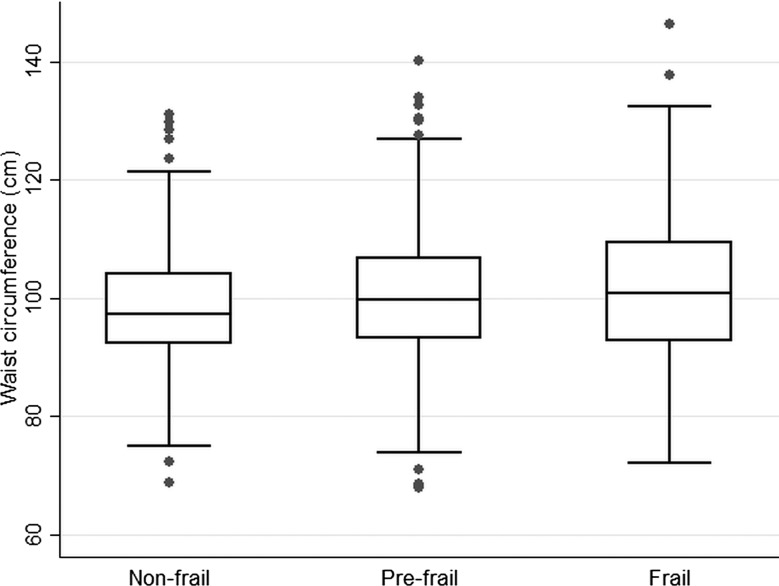
Box and whisker plot with the distribution of waist circumference according to frailty groups in 1622 older British men.

**Figure 3:**
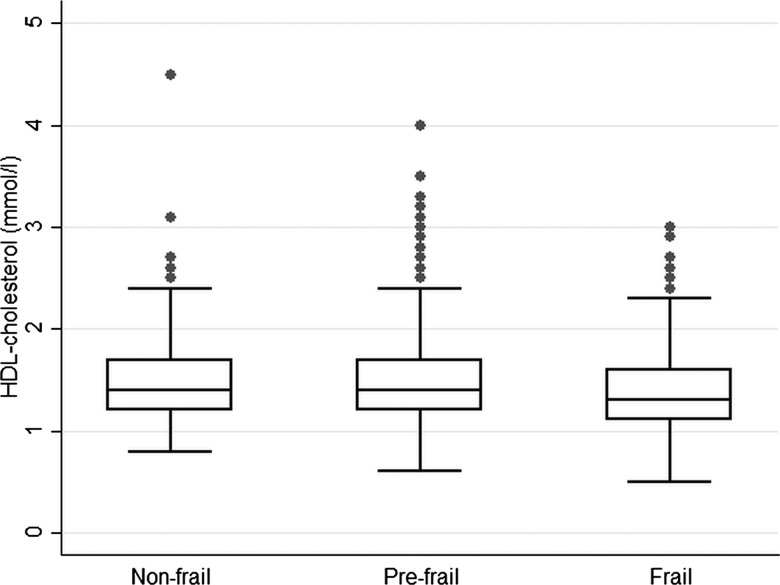
Box and whisker plot with the distribution of high-density lipoprotein (HDL) cholesterol according to frailty groups in 1622 older British men.

**Figure 4:**
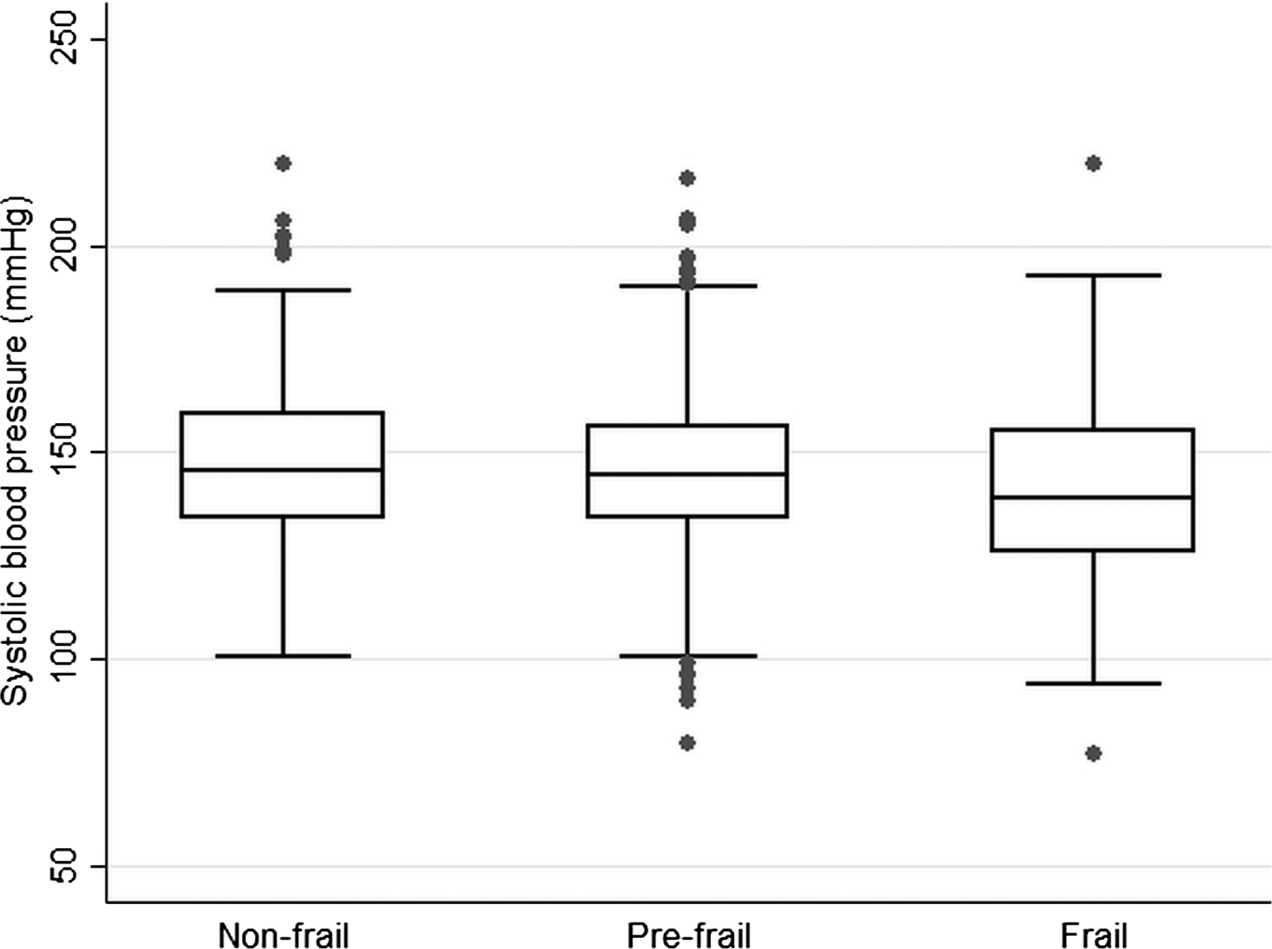
Box and whisker plot with the distribution of systolic blood pressure according to frailty groups in 1622 older British men.

Figures 1–4 present boxplots with the distribution of key cardiovascular risk factors (BMI, waist circumference, HDL-C and systolic blood pressure) according to frailty groups. Median BMI and waist circumference levels (figures 1 and 2) were higher in the frail group compared with the non-frail group (p values 0.002 and 0.0001, respectively), while HDL-C and systolic blood pressure (figures 3 and 4) were lower in those with frailty (p values 0.02 and <0.001, respectively).

Table 3 shows the associations between cardiovascular risk factors and frailty, adjusted for age, in all men. Those in the frail group were twice as likely to be obese and have high waist circumference; men in the pre-frail group also had an increased risk of being obese and having high waist circumference. Prevalence of these factors was also higher in the frail group (prevalence in frail vs non-frail group was 24% vs 16% for obesity, 46% vs 31% for high waist circumference, 20% vs 11% for low HDL-C and 78% vs 65% for hypertension). Those in the frail group had an increased risk of low HDL-C, hypertension, high heart rate, high WCC, low FEV_1_, low haemoglobin, poor renal function (low eGFR), low albumin, low ALT, high GGT, high ALP, high phosphate and low sodium. The high risk of raised GGT in the frail group remained even after adjustment for waist circumference (OR 1.85; 95% CI 1.25 to 2.74). Increased risks were also observed in the pre-frail group for some of these factors, including low HDL-C, hypertension, high WCC, low FEV_1_, poor renal function and low albumin.

**Table 3:** Vascular risk factors according to frailty in a population-based study of 1622 older British men aged 71–92 years

	Not frail	Pre-frail	Frail
Obesity			
BMI ≥30 kg/m^2^ n (%)	71 (16%)	184 (21%)	69 (24%)
OR (95% CI)	1.00	1.56 (1.15 to 2.12)	2.03 (1.38 to 2.99)
High waist circumference (>102 cm) n (%)	139 (31%)	360 (41%)	136 (46%)
OR (95% CI)	1.00	1.69 (1.32 to 2.15)	2.30 (1.67 to 3.17)
Lipid profile			
Low HDL (<1.04 mmol/L) n (%)	45 (11%)	123 (15%)	57 (20%)
OR (95% CI)	1.00	1.47 (1.02 to 2.12)	2.28 (1.47 to 3.54)
High LDL (>4 mmol/L) n (%)	48 (11%)	65 (8%)	16 (6%)
OR (95% CI)	1.00	0.66 (0.44 to 0.98)	0.48 (0.26 to 0.89)
High triglycerides (≥2.3 mmol/L) n (%)	30 (7%)	72 (9%)	28 (10%)
OR (95% CI)	1.00	1.31 (0.84 to 2.05)	1.70 (0.97 to 2.97)
Blood pressure			
Hypertension n (%)	288 (65%)	652 (74%)	235 (78%)
OR (95% CI)	1.00	1.52 (1.18 to 1.95)	1.79 (1.27 to 2.54)
Heart rate			
High heart rate (top quintile ≥77 beats/min) n (%)	67 (15%)	171 (20%)	78 (26%)
OR (95% CI)	1.00	1.34 (0.98 to 1.83)	1.90 (1.30 to 2.78)
Impaired fasting glucose			
Glucose >6.1 and <7.0 mmol/L n (%)	58 (15%)	98 (12%)	32 (12%)
OR (95% CI)	1.00	0.80 (0.56 to 1.14)	0.74 (0.46 to 1.20)
WCC			
Total WCC (top quintile ≥8.12×10^9^/L)	54 (13%)	181 (22%)	78 (28%)
OR (95% CI)	1.00	1.78 (1.28 to 2.48)	2.39 (1.60 to 3.57)
Lung function			
Low FEV_1_ (bottom quintile ≤1.91 L) n (%)	53 (12%)	155 (19%)	95 (37%)
OR (95% CI)	1.00	1.41 (1.00 to 1.99)	3.05 (2.04 to 4.54)
Low haemoglobin			
Haemoglobin (<13 g/dL) n (%)	65 (15%)	173 (20%)	117 (39%)
OR (95% CI)	1.00	1.33 (0.97 to 1.83)	3.13 (2.18 to 4.50)
Renal function			
eGFR (<60 mmL/min) n (%)	53 (13%)	192 (23%)	101 (36%)
OR (95% CI)	1.00	1.76 (1.26 to 2.46)	2.80 (1.89 to 4.15)
Albumin			
Low albumin (bottom quintile ≤42 g/L) n (%)	38 (9%)	148 (18%)	71 (25%)
OR (95% CI)	1.00	1.95 (1.33 to 2.86)	2.74 (1.76 to 4.27)
Liver enzymes			
Low ALT (bottom quintile ≤13I U/L) n (%)	58 (14%)	148 (18%)	81 (29%)
OR (95% CI)	1.00	1.16 (0.83 to 1.62)	1.87 (1.26 to 2.78)
High GGT (top quintile ≥40 IU/L) n (%)	69 (17%)	161 (19%)	75 (27%)
OR (95% CI)	1.00	1.25 (0.92 to 1.72)	2.04 (1.39 to 2.99)
Bone profile			
High ALP (top quintile ≥88 IU/L) n (%)	63 (15%)	169 (20%)	77 (27%)
OR (95% CI)	1.00	1.32 (0.96 to 1.82)	1.80 (1.22 to 2.66)
High phosphate (top quintile ≥1.21 mmol/L) n (%)	72 (17%)	173 (21%)	75 (27%)
OR (95% CI)	1.00	1.23 (0.91 to 1.68)	1.71 (1.17 to 2.50)
Sodium			
Low sodium (<138 mmol/L) n (%)	30 (7%)	79 (9%)	44 (16%)
OR (95% CI)	1.00	1.24 (0.80 to 1.94)	2.01 (1.20 to 3.36)

All ORs are age-adjusted.

ALP, alkaline phosphatase; BMI, body mass index; eGFR, estimated glomerular filtration rate; GGT, γ-glutamyltransferase; HDL, high-density lipoprotein; LDL, low-density lipoprotein; WCC, white cell count.

Table 4 presents the associations between cardiovascular risk factors and frailty specifically in 1061 men without established CVD. Among these men, frailty was associated with an increased risk of high waist circumference, low HDL-C, high heart rate, high WCC, low FEV_1_, low haemoglobin, poor renal function (low eGFR), low albumin, low ALT, high GGT, high ALP and high phosphate levels. The pre-frail group without previous CVD was also at an increased risk of having high heart rate, high WCC, low albumin and high ALP. Tests for interaction showed that the associations between cardiovascular risk factors and frailty were not significantly different in those with and without previous CVD (all p values for test for interaction were >0.05).

**Table 4:** Vascular risk factors according to frailty in 1061 older British men aged 71–92 years without established cardiovascular disease

	Not frail	Pre-frail	Frail
Obesity			
BMI ≥30 kg/m^2^ n (%)	56 (16%)	113 (20%)	25 (18%)
OR (95% CI)	1.00	1.41 (0.99 to 2.02)	1.37 (0.79 to 2.34)
High waist circumference (>102 cm) n (%)	107 (31%)	226 (40%)	62 (43%)
OR (95% CI)	1.00	1.62 (1.21 to 2.16)	2.08 (1.37 to 3.18)
Lipid profile			
Low HDL (<1.04 mmol/L) n (%)	32 (10%)	69 (13%)	30 (21%)
OR (95% CI)	1.00	1.34 (0.85 to 2.09)	2.60 (1.47 to 4.61)
High LDL (>4 mmol/L) n (%)	44 (14%)	60 (11%)	15 (11%)
OR (95% CI)	1.00	0.78 (0.51 to 1.20)	0.77 (0.40 to 1.48)
High triglycerides (≥2.3 mmol/L) n (%)	22 (7%)	52 (9%)	17 (12%)
OR (95% CI)	1.00	1.52 (0.90 to 2.57)	2.18 (1.09 to 4.36)
Blood pressure			
Hypertension n (%)	208 (61%)	380 (66%)	98 (67%)
OR (95% CI)	1.00	1.25 (0.94 to 1.66)	1.17 (0.77 to 1.79)
High heart rate			
High heart rate (top quintile ≥77 beats/min) n (%)	54 (16%)	128 (23%)	40 (27%)
OR (95% CI)	1.00	1.55 (1.08 to 2.21)	1.98 (1.22 to 3.21)
Impaired fasting glucose			
Glucose (>6.1 and <7.0 mmol/L) n (%)	41 (13%)	68 (13%)	20 (15%)
OR (95% CI)	1.00	0.90 (0.59 to 1.37)	0.91 (0.49 to 1.68)
WCC			
Total WCC (top quintile ≥8.12×10^9^/L)	37 (12%)	117 (21%)	32 (23%)
OR (95% CI)	1.00	1.94 (1.29 to 2.90)	1.93 (1.12 to 3.34)
Lung function			
Low FEV_1_ (bottom quintile ≤1.91 L) n (%)	41 (12%)	91 (17%)	42 (24%)
OR (95% CI)	1.00	1.24 (0.82 to 1.86)	2.47 (1.46 to 4.18)
Low haemoglobin			
Haemoglobin (<13 g/dl) n (%)	50 (15%)	86 (15%)	46 (31%)
OR (95% CI)	1.00	0.94 (0.64 to 1.38)	2.08 (1.28 to 3.38)
Renal function			
eGFR (<60 mmL/min) n (%)	38 (12%)	103 (19%)	45 (32%)
OR (95% CI)	1.00	1.44 (0.95 to 2.17)	2.41 (1.43 to 4.05)
Albumin			
Low albumin (bottom quintile ≤42 g/L) n (%)	30 (9%)	110 (20%)	33 (23%)
OR (95% CI)	1.00	2.16 (1.40 to 3.34)	2.26 (1.28 to 3.98)
Liver enzymes			
Low ALT (bottom quintile ≤13 IU/L) n (%)	41 (13%)	97 (18%)	45 (32%)
OR (95% CI)	1.00	1.27 (0.85 to 1.90)	2.32 (1.39 to 3.87)
High GGT (top quintile ≥40 IU/L) n (%)	55 (17%)	103 (19%)	39 (28%)
OR (95% CI)	1.00	1.16 (0.81 to 1.68)	2.03 (1.24 to 3.33)
Bone profile			
High ALP (top quintile ≥88 IU/L) n (%)	47 (15%)	115 (21%)	38 (27%)
OR (95% CI)	1.00	1.48 (1.02 to 2.16)	1.96 (1.18 to 3.26)
High phosphate (top quintile ≥1.21 mmol/L) n (%)	49 (15%)	101 (18%)	37 (26%)
Sodium			
OR (95% CI)	1.00	1.25 (0.86 to 1.83)	2.01 (1.21 to 3.33)
Low sodium (<138 mmol/L) n (%)	25 (8%)	44 (8%)	20 (14%)
OR (95% CI)	1.00	0.89 (0.53 to 1.51)	1.42 (0.73 to 2.77)

All ORs are age-adjusted.

ALP, alkaline phosphatase; ALT, alanine transaminase; BMI, body mass index; eGFR, estimated glomerular filtration rate; FEV_1_, forced expiratory volume in 1 s; GGT, γ-glutamyltransferase; HDL, high-density lipoprotein; LDL, low-density lipoprotein; WCC, white cell count.

## Discussion

This study in a representative sample of older British men aged 71–92 years shows that frailty in older age is associated with a range of cardiovascular risk factors (including dyslipidemia, obesity, poor lung function, poor renal function, raised white cells, low albumin, low sodium and altered liver function); several of these cardiovascular factors were also raised or altered in those who were pre-frail. Moreover, the association of frailty with cardiovascular risk factors was independent of established CVD. The results highlight the burden of cardiovascular risk in the frail as well as the pre-frail older population, and thus the increased risk of CVD and its complications in frail older people.

### Comparison with other studies

The results of this study present the association of frailty with a range of vascular factors in older men with and without established CVD. The results very clearly demonstrate that frailty is a condition associated with problems across multiple physiological systems. Previous studies have shown that frailty is associated with established cardiovascular risk factors such as blood pressure and HDL-C, and novel risk factors such as inflammation.^8^ ^9^ We also observed such associations of frailty with HDL-C, triglycerides and WCC. Our findings are also consistent with recent studies that show that chronic kidney disease and low ALT are associated with frailty.^8–11^ Given its association with frailty and CVD,^28^ we also investigated anaemia (low haemoglobin level) in relation to frailty. In our study, anaemia was associated with frailty, as reported in a previous study in older women.^9^ We also observed a greater risk of other factors, including low albumin and haemoglobin, in those who were frail. A particularly important aspect of our results was the finding that several of these vascular risk factors were also associated with those who were pre-frail, that is, those in the early phases of frailty. Second, these associations with vascular risk factors were found to be independent of previous CVD.

### Strengths and limitations

We present results on a range of vascular factors and their associations with frailty in a socially and geographically representative sample of older British men. We are not aware of previous studies describing associations between such a range of cardiovascular risk factors and frailty in a large population-based sample of older people. The high follow-up rate (98%) of the cohort has enabled minimal attrition rates. However, the issue of survivor bias is inevitable in cohorts of ageing populations, with frail subjects likely to have died at a higher rate. The very moderate response rate (55% for the physical examination) is likely to have selectively excluded subjects who were more frail and with a worse health profile (as observed in previous examinations).^29^ These factors are likely to have reduced the prevalence of frailty in the study population, but should not have biased the association between frailty with cardiovascular risk factors markedly. A limitation of our study is that it comprises only white European men, and the generalisability of the study to women and other ethnic groups is limited. Nevertheless, our results are comparable with other studies comprising women and non-white ethnic groups.^6^ ^9^ ^30^ We also acknowledge that our results are based on cross-sectional analyses—therefore, while we were able to demonstrate associations between risk factors and frailty, there is limited evidence for a causal association.

### Implications and conclusions

Our results highlight the adverse cardiovascular risk profile in frail older people. The presence of associations between a range of cardiovascular risk factors (not all of which are strongly age-related) and frailty reflects that the relationship between CVD and frailty is not simply due to ageing. Although the cross-sectional nature of the analyses limits understanding the direction of causality in these associations, the results demonstrate the high risk in older frail populations across markers of cardiovascular, renal, liver and haematological function—the range of markers beyond traditional cardiovascular risk factors (blood pressure, cholesterol) are indicative of poor general health across multiple physiological systems in frail older people. This highlights the high risk of CVD or its complications in frail older people—this also points to the overwhelming need to characterise and manage the high cardiovascular risk in frail older people. Cardiovascular risk factors associated with frailty and pre-frailty could be used to identify an at-risk group needing further assessment and possibly intervention aimed at cardiovascular risk prevention. At the level of the individual patient, finding one cardiovascular risk factor should prompt a review of all others, and the assessment of frailty, so that clinical care can become anticipatory. The results also demonstrate the potentially high cardiovascular risk in those who are pre-frail (or early stages of frailty), and therefore, a greater need to identify and manage frailty in its early stages in older people.

While there is an increasing recognition of frailty and its consequences in older populations, there are few initiatives implemented with a systematic management of this condition. Further research is needed to identify the extent to which interventions reducing cardiovascular risk affect frailty and its associated outcomes. A joint approach using primary prevention (early identification and management of frailty) and secondary prevention (management of vascular risk) initiatives is needed to help reduce the vascular risk and CVD-related consequences associated with frail older people.

**Key messages**

**What is already known on this subject?**

- Frailty poses an important challenge to health and social care needs of older populations in countries such as the UK and is associated with an increased risk of cardiovascular disease (CVD).
- Few population-based studies have described the cardiovascular risk profile associated with frail older people.

**What might this study add?**

- This study in a community-dwelling sample of 1622 older men shows that frailty is associated with an adverse cardiovascular risk profile (including high body mass index and waist circumference, low high-density lipoprotein-cholesterol, high blood pressure, high white cell count, poor lung function, liver function and renal function) across a range of factors affecting multiple physiological systems.
- Pre-frail groups also demonstrated some increased risk of these cardiovascular risk factors.
- The association of frailty with cardiovascular risk factors was independent of established CVD.

**How might this impact on clinical practice?**

- Frail and pre-frail older individuals need to be identified in clinical practice.
- Cardiovascular risk factors associated with frailty and pre-frailty could be used to identify an at-risk group needing further assessment and possibly intervention aimed at cardiovascular risk prevention.
- At the level of the individual patient, finding one cardiovascular risk factor should prompt a review of all others, and the assessment of frailty, so that clinical care can become anticipatory.
- Management of cardiovascular risk factors in frail and pre-frail older people could potentially reduce the burden of CVD or its complications in frail older people.
